# Perspectives on Concurrent Strength and Endurance Training in Healthy Adult Females: A Systematic Review

**DOI:** 10.1007/s40279-023-01955-5

**Published:** 2023-11-10

**Authors:** Ritva S. Mikkonen, Johanna K. Ihalainen, Anthony C. Hackney, Keijo Häkkinen

**Affiliations:** 1 Sports Technology Unit, Biology of Physical Activity, Faculty of Sport and Health Sciences, University of Jyväskylä, Kidekuja 2, 88610 Vuokatti, Finland; 2 Biology of Physical Activity, Faculty of Sport and Health Sciences, University of Jyväskylä, Jyvaskyla, Finland; 3 Department of Exercise and Sport Science, and Department of Nutrition, University of North Carolina at Chapel Hill, Chapel Hill, NC USA

## Abstract

**Background:**

Both strength and endurance training are included in global exercise recommendations and are the main components of training programs for competitive sports. While an abundance of research has been published regarding concurrent strength and endurance training, only a small portion of this research has been conducted in females or has addressed their unique physiological circumstances (e.g., hormonal profiles related to menstrual cycle phase, menstrual dysfunction, and hormonal contraceptive use), which may influence training responses and adaptations.

**Objective:**

The aim was to complete a systematic review of the scientific literature regarding training adaptations following concurrent strength and endurance training in apparently healthy adult females.

**Methods:**

A systematic electronic search for articles was performed in July 2021 and again in December 2022 using PubMed and Medline. This review followed, where applicable, the Preferred Reporting Items for Systematic Reviews and Meta-Analyses (PRISMA) guidelines. The quality of the included studies was assessed using a modified Downs and Black checklist. Inclusion criteria were (1) fully published peer-reviewed publications; (2) study published in English; (3) participants were healthy normal weight or overweight females of reproductive age (mean age between > 18 and < 50) or presented as a group (*n* > 5) in studies including both females and males and where female results were reported separately; (4) participants were randomly assigned to intervention groups, when warranted, and the study included measures of maximal strength and endurance performance; and (5) the duration of the intervention was ≥ 8 weeks to ensure a meaningful training duration.

**Results:**

Fourteen studies met the inclusion criteria (seven combined strength training with running, four with cycling, and three with rowing or cross-country skiing). These studies indicated that concurrent strength and endurance training generally increases parameters associated with strength and endurance performance in female participants, while several other health benefits such as, e.g., improved body composition and blood lipid profile were reported in individual studies. The presence of an “interference effect” in females could not be assessed from the included studies as this was not the focus of any included research and single-mode training groups were not always included alongside concurrent training groups. Importantly, the influence of concurrent training on fast-force production was limited, while the unique circumstances affecting females were not considered/reported in most studies. Overall study quality was low to moderate.

**Conclusion:**

Concurrent strength and endurance training appears to be beneficial in increasing strength and endurance capacity in females; however, multiple research paradigms must be explored to better understand the influence of concurrent training modalities in females. Future research should explore the influence of concurrent strength and endurance training on fast-force production, the possible presence of an “interference effect” in athletic populations, and the influence of unique circumstances, such as hormone profile, on training responses and adaptations.

**Supplementary Information:**

The online version contains supplementary material available at 10.1007/s40279-023-01955-5.

## Key Points

**Table:** 

Concurrent strength and endurance training generally increases parameters associated with maximal strength and endurance capacity in female participants, while several other body composition/performance benefits are reported in individual studies. Research on female athletic populations is limited.
The effects of concurrent strength and endurance training on fast-force production in female populations needs further investigation due to the importance of fast-force production in performance and functional capacity.
Menstrual status (and reasons for, e.g., menstrual dysfunction) and hormonal contraceptive use should be considered and reported in future concurrent strength and endurance training research as endocrine function or dysfunction, and related hormonal profiles, may influence acute exercise responses and subsequent training adaptations.
Most of the available studies on concurrent strength and endurance training in females are of low to moderate quality, whereas only some of the existing research reports changes in both strength and endurance parameters (rather than only strength or endurance parameters).

## Introduction

Strength and endurance training are included in global exercise recommendations [1], while a combination of strength and endurance training is often periodized and programmed to prepare for, and maintain, physical condition in competitive athletes. As such, concurrent strength and endurance training has been investigated in several populations, and an abundance of such research has been published since the classic studies of Hickson et al. in the 1980s [2, 3]. In apparently healthy adults, training strength and endurance concurrently may enhance endurance performance via improvements in force production that positively influence speed and movement economy [4–6]. It has, however, been reported that higher volumes and intensities of endurance training, particularly running, combined with strength training over prolonged periods (such as in athletes) may “interfere” with neuromuscular adaptations. It has, for example, been demonstrated that increases in maximal strength and fast-force production [7, 8] (i.e., the rate of force development or the ability of the neuromuscular system to generate force rapidly [9]) as well as muscle hypertrophy [10] may be blunted when strength and endurance are performed concurrently, although this is not always the case [11, 12]. Where evidence for “interference” of endurance training on strength, fast-force production, and hypertrophy exists in male participants [10–12], mechanisms related to acute and chronic neuromuscular fatigue, molecular pathways, and the energetic demands of concurrent training are suggested to be responsible [13]. While these mechanisms may also contribute to “interference” in females, evidence is sparse. Importantly, strength and endurance training mode, training volume, length of training period, and training session order, among other factors, modify both responses and subsequent adaptations to concurrent strength and endurance training.

Over the years, multiple review articles have been published examining concurrent strength and endurance training, including meta-analyses and systematic reviews that examine the influence of the sequence of concurrent strength and endurance training [14, 15], describe and analyze the “interference effect” [10, 16, 17], and assess the influence of training status on strength gains during concurrent strength and endurance training [18]. Several systematic reviews have also been published that are specific to concurrent strength and endurance training for optimizing endurance performance [19], rowing and canoeing [20], running [21–25], cycling [21, 26], soccer [27], multiple training modes [28], and high-intensity interval training [7, 29]. Reviews have evaluated the effect of endurance training on muscle hypertrophy [10–12], while also exploring topics such as detraining [30]. Several reviews have addressed additional mechanisms explaining the “interference effect” including signaling pathways [13, 31–36], myosin heavy chain content [36], and fiber type distribution [37]. Models for examination of the “interference effect” have been presented, while “acute” and “chronic” hypotheses behind the “interference effect” [38] have been proposed. The “acute” hypothesis suggests that residual fatigue from one training session might compromise the next training session, i.e., endurance training may affect force production in a strength training session [38]. The “chronic” hypothesis for the “interference effect” contends that muscles are subjected to competing stimuli from strength and endurance training and that attempts to adapt to both forms of training are limited due to differences in expected strength and endurance responses/adaptations [38]. While the “interference effect” has received considerable attention in review articles (and original research), as outlined above, it is generally of minimal concern for the general population for whom concurrent strength and endurance training is relatively low in volume and for whom concurrent training is recommended for health and functional performance [1].

A striking characteristic of the aforementioned reviews is that the majority do not address any unique circumstances affecting females that may influence adaptations to concurrent strength and endurance training such as the menstrual cycle, menstrual cycle dysfunction, and hormonal contraceptive use, which alter endogenous hormone profiles. The reviews that discuss sex differences or concurrent strength and endurance training specifically in females do so only briefly, although none of these review articles report specific exclusion of female-specific research. The review by Leveritt et al. (citing Bell et al., who included males and females and compared adaptations between sexes) mentions that concurrent strength and endurance training may inhibit strength development in previously trained females but not males [38, 39]. Methenitis (citing Taipale et al. and Schumann et al., both of which included males and females and compared adaptations between sexes) infers that training sequence is more important in females than in males in terms of recovery because females appear to experience higher levels of neuromuscular fatigue after endurance training [33, 40, 41]. Berryman et al. [24] noted that the possible effect of sex in adaptations to concurrent strength and endurance training could not be determined due to the limited number of studies including only females as participants. Finally, Fyfe et al. (citing Silva et al., who included only females) mention that endurance exercise at lower frequency and volume may not interfere with strength performance in physically active females [34, 42], a finding in line with research on concurrent strength and endurance training performed in males [10]. Regrettably, these reviews illustrate that the volume of concurrent strength and endurance training studies including female participants is considerably smaller than that for male participants. This markedly smaller volume of scientific research in females suggests that our knowledge regarding this topic is limited in both scope and depth. Furthermore, regarding participants of reproductive age, the possible influence of menstrual status or phase has simply not been addressed, while hormonal contraceptive use has, to our knowledge, only been addressed by Myllyaho et al. [43].

The menstrual cycle, i.e., the natural biological phenomenon (for most females) in which hormones (particularly, estradiol, progesterone, luteinizing hormone, and follicle stimulating hormone) fluctuate [44] has often been considered a “confounding factor” in sport science research. The ovarian steroids estrogen and progesterone act on several tissues and influence physiological processes throughout the female body (e.g., [45–47]). As such, it is reasonable to hypothesize that the non-reproductive functions of ovarian steroids could influence, e.g., acute responses to exercise and perhaps even longer-term adaptations. Some literature suggests that the menstrual cycle phase and accompanying hormonal concentrations can affect maximal strength [48, 49], substrate metabolism [50–52], basal body temperature [53], inflammation status [54], and protein catabolism [55]. Furthermore, it has been suggested that force production characteristics are superior during the follicular phase when estradiol and progesterone concentrations are lower [56, 57], although contrary evidence also exists [58, 59]. Combined hormonal contraceptives suppress the endogenous production of estrogen and progesterone by the hypothalamic-pituitary-ovarian axis to prevent ovulation [60] and decrease the production of testosterone and dehydroepiandrosterone (DHEA), while increasing levels of sex hormone-binding globulin (SHBG), which ultimately limits the bioactivity of these hormones. At a group level, hormonal contraceptive use may result in small decreases in exercise performance compared to eumenorrheic females [61]. Indeed, hormonal contraceptives add an additional layer of physiological complexity to research due to an abundance of different formulations and delivery methods [62–64]. It is also essential to recognize that menstrual dysfunction, which may be a result of low energy availability [65, 66] or overtraining/under recovery [67], may also significantly affect training responses and adaptations. Given that the menstrual cycle (hormonal fluctuation) and suppressed sex hormone concentrations (hormonal contraceptives and menstrual dysfunction) may influence both strength and endurance training responses/adaptations, their consideration and reporting may also be important in research regarding concurrent strength and endurance training.

Considering the present information, a systematic review and critical analysis of the literature regarding concurrent strength and endurance training specific to apparently healthy adult females are warranted in order to take steps forward in evaluating exercise prescription and programming for adult females. The purpose of this review was to (1) examine the effects of concurrent strength and endurance training on measures of strength and fast-force production as well as endurance capacity in healthy adult females; (2) identify and examine studies that take menstrual status and hormonal contraceptive use into consideration in order to determine whether these factors have been mentioned or controlled for and/or whether the influence of the menstrual cycle, menstrual status, or hormonal contraceptive use has affected study outcomes; and (3) discuss future perspectives for this area of research. It should be noted that the practical implications of concurrent strength and endurance training for healthy sedentary or physically active females versus athletic females are likely to differ, but the present review does not discriminate between these populations.

## Methods

This systematic review was performed and reported, when applicable, in accordance with the Preferred Reporting Items for Systematic Reviews and Meta-Analyses (PRISMA) [68].

### Search Strategy for Identification of Studies

A systematic electronic search for articles was performed in July 2021 using PubMed and Medline. Search terms were defined a priori and included: [“combined” OR “concurrent] AND [“strength” OR “resistance”] AND [“aerobic” OR “endurance”] AND [“training” OR “exercise”] AND [“women” OR “females”]. The lists of relevant articles and reviews obtained through the search were examined individually to identify any further studies that were subsequently added manually (see the Electronic Supplementary Material search strategy in Appendix 1). A follow-up systematic electronic search was performed in PubMed in December of 2022 to identify additional articles published later in 2021 and 2022. This review was not registered, and the review protocol was not published.

**Fig. 1:**
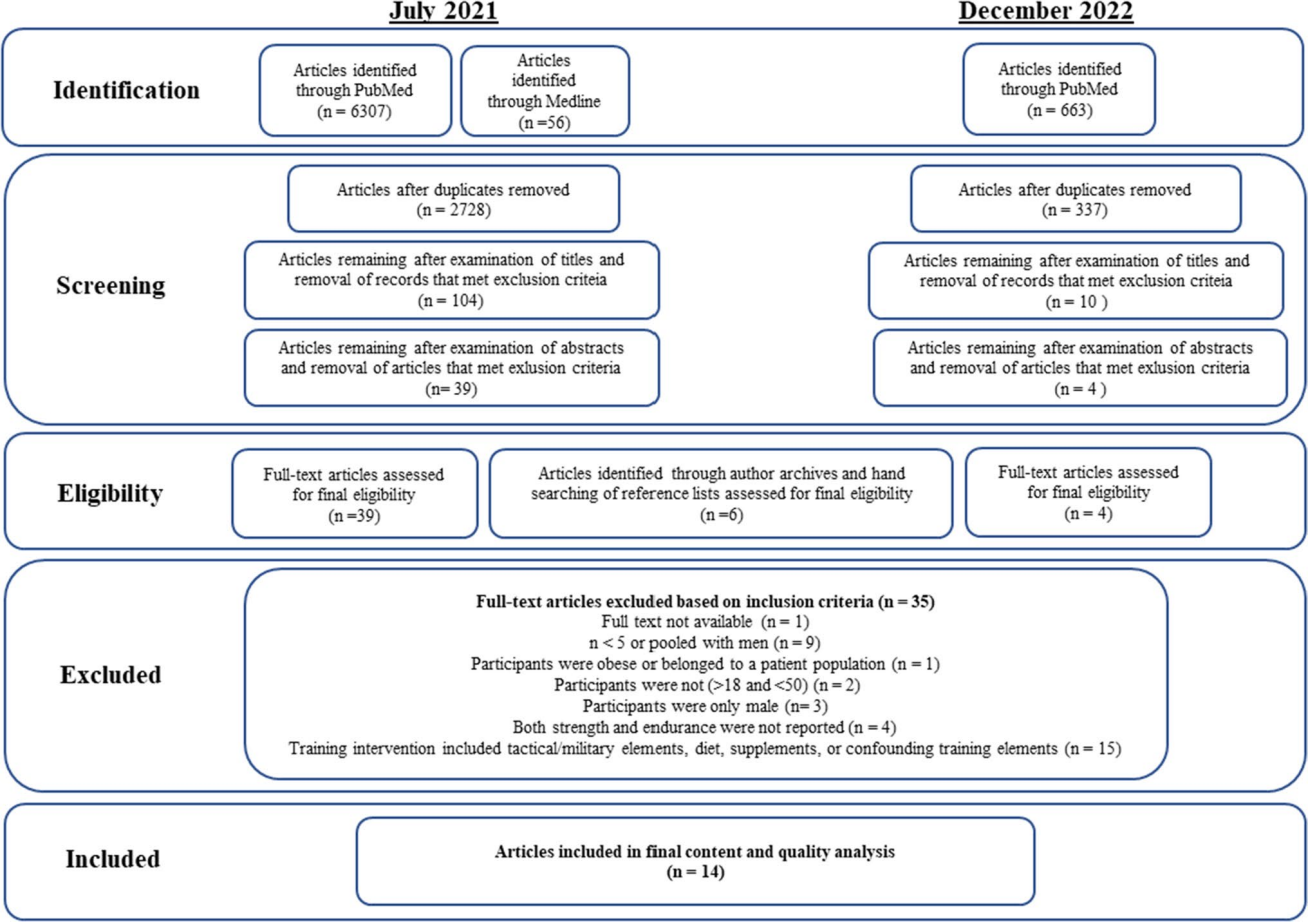
Flow chart illustrating the literature search and selection of studies

Two reviewers (RSM and JKI) independently used a two-phase screening strategy to identify relevant articles. First, the title and abstract were assessed against the predetermined inclusion and exclusion criteria described above. Studies that did not meet predetermined inclusion criteria or that met at least one of the exclusion criteria were excluded. Next, full-text articles were read and assessed against the predetermined inclusion and exclusion criteria. Conflicts were resolved by consensus with the whole group. No automation tools were used in this process. The flow chart for the literature search and selection of studies is presented in Fig. 1.

### Inclusion and Exclusion Criteria

Inclusion criteria were as follows: (1) fully published peer-reviewed publications; (2) studies published in English; (3) participants were healthy normal weight or overweight (not obese, as classified in the publication and verified < 30 kg/m^2^) females of reproductive age (mean age between > 18 and < 50) or presented as a group (*n* > 5) in studies including both females and males and where female results were reported separately; (4) participants were randomly assigned to intervention groups, when warranted, and the study included measures of maximal strength and endurance performance; where only one group was included or training groups were defined by, e.g., hormonal contraceptive use, randomization was not required; (5) the duration of the intervention was a minimum of 8 weeks to ensure a meaningful training duration likely to induce training adaptations.

Strength training was defined as weighted exercises (free weights or machines) including maximal, hypertrophic, explosive (power), and/or muscle endurance type training. Strength training classification (e.g., hypertrophic, maximal, explosive, etc.) is based on the language used by authors in their peer-reviewed publications, whereas specific loading and repetition ranges are reported in the tables later in the text. It should be noted that strength training adaptations in untrained individuals are generally both neural (muscle activation) and muscular (hypertrophy) with both submaximal and maximal loads but that trained individuals require more specific training loads to achieve desired adaptations (see, e.g., [69, 70] for more information). Endurance training was defined as continuous running, cycling, cross-country skiing, and rowing including both steady-state and interval training (excluding marching, walking, dancing, water-based exercise, and step aerobics). Study-specific endurance training volume and intensity are reported in the tables later in the text. Studies without clearly defined/described endurance training (frequency/volume, mode); those including combinations of endurance training modes with marked differences in force-production strategies, such as running and cycling; those evaluating concurrent training for rehabilitation or in populations with diseases; those including nutritional supplements or tactical military training; and/or those examining only acute responses to concurrent strength and endurance exercise/loading were excluded.

### Data Extraction and Management

Data extraction was completed by one reviewer (RSM) and was verified by one reviewer (JKI). Conflicts were resolved by consensus with the whole group. Studies were divided into groups by endurance training mode (running, cycling, or “other”) for further analysis, as endurance training mode appears to influence adaptations to strength training [10, 12]. The following information was compiled into tables that were subsequently edited and included in the “Results” section below: study hypothesis; participant information, including training status, sample size, and age; training program overview/example; main strength and endurance outcome(s); and study conclusion.

### Quality Assessment of Included Studies

Although one function of the a priori inclusion criteria was to aid in the selection of higher quality studies, the quality and bias of included studies were further assessed using a modified version of the Downs and Black checklist [71], which was specifically modified for this review, similar to [72] (see the Electronic Supplementary Material Downs and Black modified checklist in Appendix 2). The quality assessment was completed by one reviewer (RSM) and verified independently by one reviewer (JKI). Conflicts were resolved by consensus with the whole group. This checklist is made up of 15 outcomes from five domains: (1) reporting, (2) external validity, (3) internal validity—bias, (4) internal validity—confounding (selection bias), and (5) power. The maximum attainable score was 16, and study quality was categorized as follows: “high” (14–16), “moderate” (10–13), “low” (6–9), or “very low” (0–5). The results of the Downs and Black assessment were used to assign a quality rating to each study.

## Results

Fourteen studies were included in this review. It is important to note that some of the included studies appear to report findings from larger research projects, as evidenced by similar participant characteristics and training outcomes.

### Strength Training Combined with Running

Seven of the included studies examined concurrent strength and endurance running (Table 1). In this subset of studies, the duration of training interventions ranged from 8 to 16 weeks and included 2–3 strength training sessions per week. Participant training background ranged from recreationally active to competitive collegiate level athletes (tier 1 to tier 3 according to classification by McKay et al. [73]). Endurance training intensity ranged from lower intensity distance (higher volume/duration) training to higher intensity interval training as well as combinations of these training intensities. Strength training included primarily hypertrophic and heavy/maximal intensities (as described by study authors), including combinations of, e.g., maximal and explosive strength training. The quality of the studies was rated as moderate (10–13). In two studies, concurrent strength and endurance training were compared to strength training only, endurance training only, and a control group. In untrained females, ~ 8 weeks of concurrent strength and endurance running was more effective at increasing both upper and lower body strength than endurance running or no formal training (control group) and equally as effective as strength training alone. In addition, concurrent training was found to be more effective at increasing endurance capacity (measured as peak/maximal oxygen consumption [*V*O_2peak_/*V*O_2max_]) than strength training or no formal training (control group) and equally as effective as endurance training alone [74, 75].

**Table 1:** Strength training combined with running

Author, year, study design	Hypothesis	Participants (training status, sample size and age in years ± SD)	Duration of intervention and training program overview	Main strength, endurance and fast-force production outcome(s)	Study conclusion	Quality rating
Johnston et al. (1997) [76] S training was combined with E running and compared to E running only	No specific hypothesis	*Competitive distance runners* E (*n* = 6) CES (*n* = 6) Age for groups combined: (30 ± 1)	*10 weeks* E = 20–30 miles running wk^−1^ CES = E training and S training 3 × wk^−1^ Alternating program A (parallel squat, knee flexion, straight-leg heel raise, seated press, rear lateral pulldown) and B (lunge, knee extension, bent-leg heel raise, bench press, seated row, front lateral pulldown, and abdominal curl). 2–3 sets were performed with 6–20 RM	*Parallel squat (kg, mean* ± *standard error):* CES: 58.3 ± 2.8 to 81.8 ± 6.0 E: 58 ± 5 to 59.1 ± 5.2 *VO*_*2max*_* (ml kg*^*−1*^* min*^*−1*^*):* CES: 50.5 ± 2.2 to 48.0 ± 2.0 E: 51.5 ± 2.4 to 51.0 ± 1.9 *No measures of fast-force production*	S may be beneficial for improving running economy in females who have previously not participated in S	10 = Moderate
Kelly et al. (2008) [77] S training was combined with E running and compared to E running only	CES training involving a heavy S training protocol will result in improvements in E running performance when compared with E training alone	*Recreational E runners* CES (*n* = 7, 21 ± 2) E (*n* = 9, 20 ± 4)	*10 weeks* CES = 3 × wk^−1^ with 8 h rest between E and S where S = 3 × 5 reps progressive overload of squats, calf raises, hip extension, hip flexion, hamstring curl, seated row, bench press, and core exercises E = continuous, long, slow distance and intervals	*1-RM squat (kg):* CES: 67.6 ± 15.9 to 79.7 ± 12.0 E: 66.2 ± 15.9 to 62.2 ± 17.3 *VO*_*2peak*_* (ml kg*^*−1*^* min*^*−1*^*):* CES: 39.9 ± 5.2 to 45.1 ± 7.2 E: 39.5 ± 6.0 to 42.3 ± 4.9 *No measures of fast-force production*	CES led to statistically non-significant improvements in 3-km running and improved S of lower extremities with no differences observed between CES and E only	12 = Moderate
Hendrickson et al. (2010) [74] Concurrent S and E training was compared to S only, E only, and recreationally active C groups	Because tactical occupations require maximum S, muscle E, and aerobic E, participating in concurrent training will not interfere with improvements in physical capacities and will improve performance more than performing single-mode S or E training	*Recreationally active females* CES (*n* = 15, 20 ± 0) S (*n* = 18, 21 ± 1) E (*n* = 13, 21 ± 0) C (*n* = 10, 20 ± 1)	*12 weeks* CES = both the resistance and E below S = non-linear periodized training 3 × wk^−1^ (light 12 RM to heavy 3–5 RM) (depending on day = squat/leg press/deadlift, bench press, lateral pull-down, upright row/high pull, calf exercises, abdominal work, shoulder press/push press, seated row, incline bench press) E = continuous running and sprint intervals of various kinds 3 × wk^−1^ C = no formal training	*1-RM squat (kg):* CES: 38.2 ± 1.2 to 41.5 ± 1.2 S: 38.2 ± 1.1 to 38.9 ± 1.1 E: 40.0 ± 1.3 to 42.4 ± 1.3 C: 38.0 ± 1.5 to 38.3 ± 1.4 *VO*_*2peak*_* (ml kg*^*−1*^ *min*^*−1*^*):* CES: 38.2 ± 1.2 to 41.5 ± 1.2 S: 38.2 ± 1.1 to 38.9 ± 1.1 E: 40.0 ± 1.3 to 42.4 ± 1.3 C: 38.0 ± 1.5 to 38.3 ± 1.4 *Squat jump, peak power (W):* CES: 1341.9 ± 88.3 to 1652.9 ± 117.5 S: 1355.7 ± 74.2 to 1755.5 ± 98.7 E: 1378.7 ± 81.9 to 1580.5 ± 108.8 C: 1355.2 ± 96.8 to 1588.0 ± 128.7	CES and S increased maximal S and power. Similar increases in aerobic capacity between CES and E	13 = Moderate
Nindl et al. (2010) [75] Concurrent S and E training was compared to S only, E only, and recreationally active C groups	Concurrent S and E training might result in greater perturbations in the IGF-I system compared to single modes of exercise (S and E). IGF-I bioactivity will be superior to immunoreactive IGF-I in reflecting training-associated fitness improvements	E (*n* = 13) S (*n* = 18) ES (*n* = 15) C (*n* = 10) Age for groups combined: 25 ± 5	*8 weeks* S = hypertrophic (light), moderate, and maximal (heavy) days 3 × wk^−1^ E = running 3 × wk^−1^ including continuous running (20–30 min at 70–85% HR_max_) and sprint-type interval training (400-, 800-, 1200-, 1600-m runs with equal recovery) ES = both programs (S + E performed together on 3 days wk^−1^) C = no formal training	*1-RM back squat (kg):* CES: 57.2 ± 3.0 to 77.1 ± 1.1 S: 53.8 ± 2.7 to 80.7 ± 2.7 E: 53.8 ± 3.0 to 61.4 ± 1.0 C: 61.6 ± 3.5 to 68.2 ± 3.5 *VO*_*2peak*_* (ml* *kg*^*−1*^ *min*^*−1*^*):* CES: 38.2 ± 1.5 to 41.5 ± 1.2 S: 38.2 ± 1.1 to 38.9 ± 1.1 E: 40.0 ± 1.3 to 42.4 ± 1.4 C: 38.0 ± 1.5 to 38.3 ± 1.4 *No measures of fast-force production*	Favorable adaptations were observed in S, aerobic fitness, and body composition. Circulating IGF-I was negatively associated with body fat and positively associated with measures of aerobic fitness and muscular E. Circulating IGF-I was not associated with measures of fat-free mass or muscle S	12 = Moderate
Barnes et al. (2013) [79] Different S training modes were combined with E training and compared	No specific hypothesis	*Collegiate cross-country runners* Traditional HRT: Males (*n* = 13, 20 ± 1) Females (*n* = 19, 20 ± 1) PRT: Males (*n* = 10, 21 ± 1) Females (*n* = 10, 21 ± 1)	*9 weeks (7–10 weeks)* E = runners maintained their normal E training S = 2 × wk^−1^ over a 7- to 10-week period (with exceptions to weeks 10, 12, and 13, where only 1 session was performed); HRT or PRT matched for volume load. Each session included 4 lower body lifts or 4 complex set lifts (lower body lift followed by plyometric exercise) as well as upper body lifts	*1-RM leg press (determined from 3- to 6-RM test):* HRT females: 35.9 ± 2.3 (change score = 44.5 ± 10.3) PRT females: 41.2 ± 8.0 (change score = 29.6 ± 8.7) HRT males: 70.7 ± 13.3 (change score = 31.1 ± 3.5) PRT males: 68.7 ± 13.6 (change score = 24.3 ± 5.6) *VO*_*2max*_* (ml* *kg*^*−1*^ *min*^*−1*^*):* HRT females: 52.3 ± 3.3 (change score = 3.4 ± 6.3) PRT females: 51.3 ± 2.8 (change score = 4.7 ± 5.2) HRT males: 63.7 ± 4.7 (change score = 1.2 ± 7.1) PRT males: 63.8 ± 4.6 (change score = 0.1 ± 5.2) *5-jump (straight-leg) plyometric jump test peak force (Nkg*^*−1*^*):* HRT: 64.9 ± 14.8 (change score = 7.5 ± 14.8) PRT: 70.7 ± 14.3 (change score = 1.1 ± 14.3)	Both HRT and PRT had beneficial effects on competition times in females, but effects were possibly harmful in males. PRT was possibly harmful to cross-country competition performance and laboratory measures when compared to HRT. Females should include HRT in season, males should proceed with caution Male and female HRT showed greater improvements in running economy compared with PRT	10 = Moderate
Taipale et al. (2014) [78] A mixture of maximal and explosive S training was compared to S training combined with muscle E exercise	Mixed maximal and explosive S training will be more effective than body weight circuit training for improving neuromuscular characteristics of lower extremities that will also have a small influence on E performance characteristics	*Recreational E runners* CES females (*n* = 9, 29 ± 7) C females (*n* = 9, 35 ± 6) CES males (*n* = 9, 31 ± 9) C males (*n* = 7, 34 ± 9)	*16 weeks (8 weeks preparatory* + *8 weeks intervention)* S = 50–70% loads for 12 sessions for 8 wk followed by 2 sets of 6 RM progressing to 3 sets of 4-RM squat and leg press and body weight (explosive) box jumps and vertical jumps (+ core) 2 × wk^−1^ for 8 wk C = 50–70% loads for 12 sessions for 8 wk followed by circuit training with work:rest ratio of 45 s:15 s and 50 s:10 s was used during wk 8–12 and 12–16	*1-RM leg press:* CES females: + 14% C females: + 7% CES males: + 6% C males: + 6% *VO*_*2max*_* (ml* *kg*^*−1*^ *min*^*−1*^*):* CES females: 43.7 ± 2.4 to 45.4 ± 2.7 C males: 45.7 ± 3.0 to 49.8 ± 7.0 (No significant increases in C females or CES males) *Countermovement jump:* CES females + 11% CES males + 11% (+ 9% C males and + 7% in C females)	Improvements in explosive S, muscle activation, and maximal S appear to, in combination with low-volume/intensity E, enhance peak running speed and submaximal running characteristics. Females had a greater relative increase in maximal S while males appeared to make more systematic improvements in submaximal running characteristics. Submaximal heart rate and blood lactate improved more in CES females than in C females	11 = Moderate
Myllyaho et al. (2018) [43] The possible effects of hormonal contraceptives on training adaptations were examined in two groups performing high-intensity S and E were combined	Hormonal contraceptive use may impair improvements in S and E performance as well as muscle hypertrophy and fat loss	*Recreationally active females* CES HC (*n* = 9, 28.2 ± 3.1) CES NHC (*n* = 9, 31.3 ± 5.4)	*10 weeks* CES = 2 × wk^−1^ 1 session of 4 × 4-min running intervals progressing from + 70% to 90% of HR_max_ and 1 sprint training session with 3 × 3 × 100-m all-out sprints combined with 2 × wk^−1^ S with progressively increasing loads (50% to 85% 1 RM). Main exercises included maximal and explosive sets of bilateral squats, bilateral leg press, knee flexion, calf raise, and calf jump (2–3 sets with 6–10 reps set^−1^)	*1-RM leg press (kg):* CES HC: 114 ± 15 to 124 ± 16 CES NHC: 118 ± 18 to 128 ± 21 *3000-m running time:* CES HC: improved 3.5 ± 4.5% CES NHC: improved 1.0 ± 3.3% *Countermovement jump (cm):* CES HC: 25.8 ± 3.0 to 27.1 ± 4.2 CES NHC: 26.2 ± 4.9 to 29.0 ± 4.5	No significant differences in adaptations between HC and non-users in terms of adaptations to CES training	13 = Moderate

Values presented as mean ± SD unless otherwise noted

*C* control, *CES* concurrent strength and endurance training in which the order of training is not specified or relevant to the research question, *E* endurance, *HC* hormonal contraceptive users, *NHC* naturally menstruating females (i.e., not using hormonal contraceptives), *HRT* heavy resistance training, *HR*_*max*_ maximal heart rate, *IGF-1* insulin-like growth factor 1, *PRT* plyometric and heavy resistance training, *reps* repetitions, *RM* repetition maximum, *S* strength, *SD* standard deviation, *VO*_*2max*_ maximal oxygen uptake (presented relative to body mass in milliliters per kilogram per minute), *VO*_*2peak*_ peak oxygen consumption (presented relative to body mass in milliliters per kilogram per minute), *wk* week(s)

In two studies, strength training was added to endurance running. In competitive runners, improvements in running economy were attributed to strength training, while maximal aerobic capacity (*V*O_2max_) remained unchanged after 10 weeks of concurrent strength and endurance running versus running alone [76]. In recreational runners, no significant differences between concurrent strength and endurance running and endurance running only groups were reported in *V*O_2max_, running economy, or body composition adaptations [77].

One study compared strength training versus body weight (muscle endurance) training, and a combination of maximal and explosive strength training performed concurrently with endurance running in recreational runners appeared to be superior to endurance running performed concurrently with body weight (muscle endurance) training for increasing maximal strength; however, improvements in peak running speed were similar between groups [78]. Another study compared adaptations between recreationally trained naturally menstruating females and recreationally trained females using hormonal contraceptives. This study indicated that 10 weeks of high-intensity, different-day concurrent strength and endurance running improved maximal strength of the leg extensors and countermovement jump height, whereas no differences between groups were observed in 3000-m running time improvements [43].

Four of the above studies [43, 74, 78, 79] included assessment of fast-force production using either countermovement jump, squat jump, or 5-jump (straight-leg) plyometric jump test peak force. Mixed maximal and explosive strength training performed concurrently with endurance running [43, 78] improved countermovement jump, while non-linear periodized strength training (with loads ranging from 3 repetitions maximum [RM] to 12 RM) performed concurrently with aerobic training increased squat jump [74]. Heavy resistance training appeared to be more effective at improving force production in a 5-jump plyometric test than volume-load–matched plyometric training, which could be relevant in terms of force production for running performance [79].

### Strength Training Combined with Cycling

Four of the included studies examined concurrent strength and endurance training in which the endurance training mode was cycling (Table 2). In this subset of studies, the duration of the training intervention ranged from 9 to 24 weeks and included 1–2 strength training sessions per week. Participant training background ranged from untrained/sedentary to recreationally active (tier 0 to tier 1 according to classification by McKay et al. [73]). Endurance training intensity ranged from low-intensity distance training to high-intensity interval training including combinations and progressions of these training intensities. Strength training intensity ranged from muscle endurance to hypertrophic and heavy/maximal strength training in addition to combinations and progressions of these strength training modes. The quality of the studies was rated as moderate (10–11).

**Table 2:** Strength training combined with cycling

Author, year, study design	Hypothesis	Participants (training status, sample size and age in years ± SD)	Duration of intervention and training program overview	Main S, E, and fast-force production outcome(s)	Study conclusion	Quality rating
Schumann et al. (2015) [81] Order of S and E training in a single session was compared with performing S and E on separate days. Adaptations in females and males were compared	Starting concurrent training sessions with S training (rather than E training or performing S and E on separate days) may lead to compromised cardiorespiratory adaptations	*Physically active females and males* ES females (*n* = 15, 30 ± 5) SE females (*n* = 13, 29 ± 4) DD females (*n* = 18, 30 ± 8) ES males (*n* = 15, 30 ± 5) SE males (*n* = 13, 29 ± 4) DD males (*n* = 18, 30 ± 8)	*24 weeks* ES, SE, and DD = 2 × wk^−1^ during wk 1–12 and 2–3 × wk^−1^ during wk 13–24 E = low to moderate steady-state intensity progressing to high-intensity interval sessions including steady-state cycling and high-intensity interval sessions S = bilateral dynamic horizontal leg press, bilateral and unilateral dynamic knee extension and flexion + dynamic seated vertical press and lateral pulldown, crunches, torso rotation, and lower back extension. Starting with 2–4 sets of 15–20 reps at 40–60% 1 RM with 1-min inter-set rest to 2–5 sets of 3–5 reps at 85–95% of 1 RM with 3-min inter-set rest	*1-RM leg press (kg):* ES females: 102 ± 22 to 115 ± 23 SE females: 100 ± 18 to 116 ± 17 DD females: 88 ± 12 to 106 ± 14 ES males: 157 ± 30 to 175 ± 27 SE males: 143 ± 23 to 166 ± 20 DD males: 142 ± 24 to 159 ± 22 *VO*_*2peak*_* (ml* *kg*^*−1*^ *min*^*−1*^*):* ES females: 30.7 ± 3.8 to 34.0 ± 4.0 SE females: 33.8 ± 4.7 to 36.9 ± 4.8 DD females: 27.9 ± 5.8 to 34.7 ± 5.8 ES males: 42.2 ± 7.2 to 44.6 ± 5.1 SE males: 42.5 ± 7.0 to 45.3 ± 6.9 DD males: 36.2 ± 6.5 to 42.4 ± 6.6 *No measures of fast-force production*	Training S and E on different days may be superior for improving *V*O_2peak_ in both males and females. Females may benefit from performing E prior to S due to superior improvements in submaximal *V*O_2_	11 = Moderate
Eklund et al. (2016) [82] Order of S and E training in a single session was compared with performing S and E on separate days. Adaptations in females and males were compared	No specific hypothesis	*Previously untrained females and males* ES females (*n* = 17, 29 ± 6) SE females (*n* = 15, 29 ± 4) DD females (*n* = 18, 30 ± 8) ES males (*n* = 17, 30 ± 6) SE males (*n* = 18, 30 ± 4) DD males (*n* = 21, 30 ± 6)	*24 weeks* ES, SE, and DD = 2 × wk^−1^ during wk 1–12 and 2–3 × wk^−1^ during wk 13–24 E = low to moderate steady-state intensity progressing to high-intensity interval sessions including steady-state cycling and high-intensity interval sessions S = bilateral dynamic horizontal leg press, bilateral and unilateral dynamic knee extension and flexion + dynamic seated vertical press and lateral pulldown, crunches, torso rotation, and lower back extension. Starting with 2–4 sets of 15–20 reps at 40–60% 1 RM with 1-min inter-set rest to 2–5 sets of 3–5 reps at 85–95% of 1 RM with 3-min inter-set rest	*1-RM leg press:* See Schumann et al. 2015 [81] *VO*_*2max*_*:* See Schumann et al. 2015 [81] *No measures of fast-force production*	All 3 CES training modes led to significant increases in S and E performance as well as lean body mass. Decreased body fat mass was observed only in DD	11 = Moderate
Eklund et al. (2016) [83] Order of S and E training in a single session was compared	No specific hypothesis	*Previously untrained females* ES (*n* = 15, 29 ± 6) SE (*n* = 14, 29 ± 4)	*24 weeks* 2 × wk^−1^ training + 12 wk of 5 × / 2 wk training S = (2–4 sets of 15–20 reps at ~ 60% 1 RM) + (2–5 × 8–12 at 80–85% of 1 RM, 1–2 min rest) + (2–5 × 3–5 at 85–95% of 1 RM, 3–4 min rest) of horizontal leg press, seated hamstring curls, and seated knee extensions + upper body and trunk E = low to moderate steady-state intensity progressing to high-intensity interval sessions including steady-state cycling and high-intensity interval sessions	*1-RM leg press:* See Schumann et al. 2015 [81] *W*_*max*_ *(significant in both groups):* ES by 21 ± 10% from 170 ± 26 W SE by 16 ± 12% from 182 ± 27 W *No measures of fast-force production*	S, E performance and muscle CSA increased similarly regardless of training order over 24 wk. Previously untrained females can improve performance and increase muscle CSA utilizing either exercise order	11 = Moderate
Kyröläinen et al. (2018) [80] Concurrent S and E training was investigated in a population of untrained females	Physical fitness will improve, and some health biomarkers will change positively, while no changes were expected in body composition due to low training volume	*Previously untrained females* CES (*n* = 17, 27 ± 2)	*9 weeks* CES = 7 weeks of 2 S + 1 E followed by 2 wk of 2 E + 1 S, where E = indoor cycling starting with 30 min and progressing to 55 min at intensity of 85–91% *V*O_2max_ and S = 5–15 reps of leg press, knee extension and flexion, toe rise, lateral pulldown, bench press, biceps curl, triceps curl, back extension, and abdominal curl	*Maximal isometric leg press:* CES: 1911 ± 182 to 2464 ± 240 N (28.9%) *VO*_*2max*_* (ml* *kg*^*−1*^ *min*^*−1*^*):* CES: + 8.5% *Rate of force development was unchanged*	CES can induce significant S, E, and health benefits such as improvements in total cholesterol	10 = Moderate

Values presented as mean ± SD unless otherwise noted

*CES* concurrent strength and endurance training in which the order of training is not specified or relevant to the research question, *CSA* cross-sectional area, *DD* strength and endurance training performed on different days, *E* endurance, *ES* endurance before strength training on the same day, *reps* repetitions, *RM* repetition maximum, *S* strength, *SD* standard deviation, *SE* strength before endurance on the same day, *VO*_*2max*_ maximal oxygen uptake (presented relative to body mass in milliliters per kilogram per minute), *VO*_*2peak* _peak oxygen consumption (presented relative to body mass in milliliters per kilogram per minute, *W*_*max*_ maximal workload, *W* watts, *wk* week(s)

One study examined low-volume strength training performed concurrently with endurance cycling within a single group. Untrained participants improved isometric strength of the leg and arm extensors and maximal aerobic capacity as well as blood lipid profile [80]. Three studies examined longer-term periodized concurrent strength and endurance cycling interventions where training order (endurance before strength, strength before endurance, or alternate day training) was a central theme. All three training orders induce favorable changes in maximal strength of the lower extremities, endurance capacity, and body composition [81–83]. Performing strength and endurance training on different days was, however, suggested to have additional advantages in terms of improving endurance performance and decreasing fat mass due to a potential for increased daily physical activity that may accrue via warm-ups and cool-downs as well as commuting to the gym (possibly walking or biking). Interestingly, these studies suggest that endurance (cycling) training before strength training may be more effective for improving submaximal endurance performance in females than in males although marked differences in maximal strength development of the lower extremities were not observed. [78]

Only one study examined fast-force production [80], reporting no change in the rate of force development analyzed from maximal isometric leg press.

### Strength Training Combined with Other Forms of Endurance Training

Three of the included studies examined concurrent strength and endurance training in which the endurance training mode was either rowing or cross-country skiing (Table 3). In this subset of studies, intervention length ranged from 9 to 16 weeks and included 2–3 strength training sessions per week. Participant training background ranged from recreationally active to trained cross-country skiers (tier 1 to tier 3 according to classification by McKay et al. [73]). Endurance training in the studies where the endurance training mode was rowing was completed at a heart rate equivalent to the ventilation threshold 3–4 times per week [39, 84], whereas the cross-country skiing was performed primarily at lower intensities but also included higher intensity interval training [85]. Strength training in these studies included heavy/maximal strength training. The quality of the studies was rated as low to moderate (9–10).

**Table 3:** Strength training combined with rowing/cross-country skiing

Author, year, study design	Hypothesis	Participants (training status, sample size and age)	Training program overview/example	Main S and E outcome(s)	Study conclusion	Quality rating
Bell et al. (1997) [39] Concurrent S and E training were compared to S training only in females and males	Concurrent S and E training may induce a greater catabolic state than S training alone	*Rowers and students* S females (*n* = 6, 21 ± 3) CES females (*n* = 8, 24 ± 5) S males (*n* = 8, 24 ± 6) CES males (*n* = 14, 23 ± 2) *Results presented separately*	*16 weeks* S = 3 × wk^−1^ using free weights and machines including bilateral incline leg press, knee extension, knee flexion, bench press, seated row, lateral pulldowns, and arm curls. Abdominal curl-ups and stretching included. Volume and intensity were progressively overloaded every 4 wk using computer software (3–6 sets, 2–10 reps, 65–85% intensity) E = 3 × wk^−1^ where 2 sessions on a rowing machine starting at 30 min and progressing 5 min every 4 wk at an HR equivalent to ventilation threshold and 1 session included intervals: 5 × 3 min with 3 min active recovery adding 1 additional set every 4 weeks. Interval intensity was 90% *V*O_2max_	*Bilateral incline leg press 1 RM (kg):* S females: 152.8 ± 20.2 to 275.9 ± 38.5 CES females: 219.9 ± 14.1 to 263.7 ± 17.9 S males: 260.5 ± 22.3 to 351.3 ± 23.5 CES males: 305.2 ± 18.9 to 441.2 ± 20.9 *Rowing test* *VO*_*2max*_* (L* *min*^*−1*^*):* CES females: 2.96 ± 0.27 to 3.08 ± 0.29 CES males: 4.27 ± 0.5 to 4.38 ± 0.51 *No measures of fast-force production*	A difference in time-course of adaptations was observed between males and females, but no significant differences in increases in S or E performance were observed. CES may inhibit S development in previously trained females but not in males. Increases in *V*O_2max_ and power output at ventilation threshold as well as S were observed. Females had elevated urinary cortisol in both S and CES, but no changes in testosterone were observed	9 = Low
Haykowsky et al. (1998) [84] Concurrent S and E training adaptations were compared between females and males	E and S training will increase left ventricular wall thickness, diastolic cavity dimension, estimated absolute and relative left ventricular mass	*Novice and experienced collegiate rowers* CES females (*n* = 17, 23 ± 5) CES males (*n* = 8, 23 ± 6)	*10 weeks* S = 2 × wk^−1^ 65% to 85% 1 RM (2–6 reps, 3–6 sets) incline leg press, knee extension and flexion, bench press, seated row, lateral pulldowns, and arm curls (abdominal curls and stretching) E = 4 × wk^−1^ rowing 3 sessions wk^−1^ were just below VT 40 to 70 min plus 1 interval session wk^−1^ at intensity of 90% *V*O_2max_ 2 min "on" 2 min recover starting at 5 reps and progressing to 10	*Leg press 1 RM (kg):* CES females: 171.6 ± 38.5 to 246.3 ± 45 CES males: 294.9 ± 59.1 to 365.3 ± 70.7 *Rowing test VO*_*2max*_* (L* *min*^*−1*^*):* CES females: 2.96 ± 0.37 to 3.17 ± 0.39 CES males 4.35 ± 0.65 to 4.47 ± 0.62 *No measures of fast-force production*	Changes were observed in *V*O_2max_, muscular S and rowing performance as well as left ventricular systolic function and morphology	9 = Low
Hoff et al. (1999) [85] Heavy upper body S training was added to E training in female cross-country skiers	Maximal S training will improve double-pole performance (improved work economy and anaerobic threshold), and work economy will improve due to a reduction in relative workload (% 1 RM) and time to peak force during double poling at maximal aerobic velocity	*Cross-country skiers* CES (*n* = 8, 17.8 ± 0.4) E (*n* = 7, 18.0 ± 0.4)	*9 weeks* CES = S 3 d·wk^−1^ (3 × 6 RM on a modified cable pulley for upper body) + E volume 8.5 ± 0.8 h wk^−1^ (running and skiing) E = 9.2 ± 1.2 h wk^−1^ (running and skiing)	*Double-pole 1 RM:* CES: 14.5% ± 1.8% E: no change *Double pole VO*_*2max*_* (ml* *kg*^*−1*^ *min*^*−1*^*):* CES: 46.5 ± 1.5 to 48.6 ± 2.2 E: 46.7 ± 2.1 to 50.8 ± 2.0 *No measures of fast-force production*	Upper body maximal S improves double-poling performance via improved work economy. Time to peak force in double poling improved. Improvements happened even with a relatively high volume of E	10 = Moderate

Values presented as mean ± SD unless otherwise noted

*CES* concurrent strength and endurance training in which the order of training is not specified or relevant to the research question, *d* days, *E* endurance, *HR *heart rate, *reps* repetitions, *RM* repetition maximum, *S* strength, *SD* standard deviation, *VO*_*2max*_ maximal oxygen uptake (presented relative to body mass in milliliters per kilogram per minute or as liters per minute), *VT* ventilatory threshold, *wk* week(s)

Two studies combined rowing with whole body strength training with a focus on measuring the lower extremities [39] (and left ventricular morphology [84]). One study examined concurrent strength and endurance (ski ergometer) training in the upper body, where both testing and training were targeted at the upper body [85]. Understandably, the study participants were trained cross-country skiers that were accustomed to using their upper bodies as part of their sport. The investigation revealed improved upper body strength and significantly greater time to exhaustion. Overall, these three studies indicated that concurrent rowing or cross-country skiing and strength training appeared to increase 1-RM strength (bilateral leg press or double pole) and endurance performance/capacity, whereas only Bell et al. [39] suggested endurance training may inhibit strength development (measured as bilateral incline leg press) in previously trained females. Regrettably, fast-force production was not assessed in any of the included studies.

### Fast-Force Production

Only five of the 14 included studies incorporated jumping tests (e.g., squat jump, peak power, countermovement jump, and straight-leg 5-jump) to evaluate fast-force production. Overall, these studies indicate that strength training combined with endurance training improves measures of fast-force production. Of note is that all of the included studies indicating increases in fast-force production trained strength concurrently with running rather than cycling. Squat jump peak power increased by ~ 23% and ~ 29% in concurrent strength and running and strength only training, respectively, while it only increased by ~ 15% and ~ 17% in endurance training only and control groups [74]. An ~ 11% improvement in countermovement jump height was observed in a group performing a mixture of maximal and explosive strength training combined with running, where the relative increase in performance of the group that completed mixed maximal and explosive strength training combined with endurance running was greater than the control group that combined endurance running with body weight circuit training [78]. In contrast, improvements in peak force from a straight-leg 5-jump plyometric jump test (Nkg^−1^) were larger in a group combining running with heavy resistance training than a group performing volume-matched plyometric and heavy resistance training (change score of 7.5 ± 14.8 vs. 1.1 ± 14.3) [79].

Two studies examined fast-force production from a different perspective. Myllyaho et al. [43] reported a greater increase in countermovement jump height in naturally menstruating females (11%) compared with hormonal contraceptive using females (4%), although the difference between groups was not statistically significant. Kyröläinen et al. [80] reported that isometric rate of force development was unchanged in a single group performing concurrent strength and endurance training, but the lack of a single-mode control group makes it impossible to determine whether this was a result of training strength and endurance concurrently or simply a function of the prescribed training not improving rate of force development.

### Menstrual Status and Hormonal Contraceptive Use

A limited subset of studies reported, or took into consideration, menstrual status or hormonal contraceptive use in terms of timing of testing/measurements or during recruitment and subsequent data analyses. Nindl et al. [75] and Hendrickson et al. [74] reported that their investigation included “regularly menstruating” females, and the authors indicated that blood draws were always at the same (unreported) time (phase) of the menstrual cycle for analysis of serum hormones. Kyröläinen et al. [80] reported that none of the participants were using hormonal contraceptives, and Eklund et al. [83] reported that hormonal contraceptive users and non-users were included in the same groups. Kyröläinen et al. [80] and Eklund et al. [83] did not time testing according to menstrual cycle phase. Of the included studies, only Myllyaho et al. [43] considered both menstrual cycle status and hormonal contraceptive use during recruitment and subsequently compared a group of females using monophasic oral contraceptives and self-reported eumenorrheic/naturally menstruating females. Reported weaknesses in this study were the inclusion of several formulations of monophasic combined oral contraceptives in addition to a lack of hormonal verification throughout the study (including ovulation). Nevertheless, levels of progesterone and estrogen were analyzed from blood serum in an effort to confirm that performance testing was completed in the early follicular phase (days 1–5 of the menstrual cycle) [43].

### Methodological Quality

**Fig. 2:**
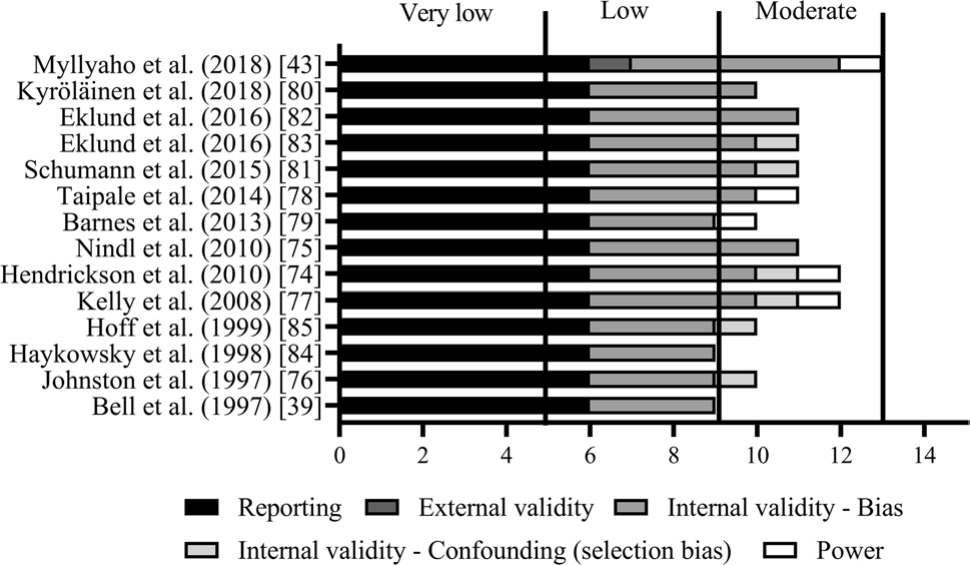
Quality ratings of the included studies according to a modified Downs and Black checklist

The methodological quality of the selected studies is presented in Fig. 2. Overall study quality was low to moderate (9–12), with an average rating of 10.7, which is at the lower end of the “moderate” study quality classification. Several studies lacked power calculations and/or did not report compliance with the intervention or whether or not participants were randomized into intervention groups, and the majority of studies did not consider hormonal status. The majority of studies lacked external validity and had problems regarding internal validity in terms of selection bias.

## Discussion

During the last few decades, there has been a dramatic increase in the number of females participating in both recreational physical activity and the highest levels of elite sport [86]. Regrettably, training and performance research in females has not kept pace with this exponential rise in participation [87]. As both strength and endurance training are a vital part of training for both recreationally active and competitive females, a review of the current literature is warranted. The studies included in this review generally reported that concurrent strength and endurance training is an effective training strategy for females due to observed gains in maximal strength that were sometimes also accompanied by increases in endurance capacity/performance in addition to other parameters associated with health, including body composition and blood lipid profile. Most study designs, however, did not allow us to determine whether concurrent strength and endurance training is more (or less) effective than strength or endurance training alone for developing, e.g., maximal strength, fast-force production, or endurance capacity/performance. There is insufficient evidence (particularly from high-quality studies) to form a conclusion regarding the “interference effect” of endurance on maximal strength and/or fast-force production in females, although “interference” may be a concern when combining training modes in athletes. In this review, we noted that research on concurrent strength and endurance training in females has rarely considered menstrual status or hormonal contraceptive use, factors that may influence study outcomes. Ultimately, the limited number of high-quality studies regarding concurrent strength and endurance training in female populations, and especially athletic populations [18], suggests that more scientifically sound research in females is needed.

### Concurrent Strength and Endurance Training Approaches and Outcomes

Included studies reported training interventions ranging widely from 7 to 24 weeks in populations that ranged from untrained to trained according to the classification by McKay et al. [73]. Included studies generally incorporated linear training progression in terms of intensity and volume, but did not necessarily utilize a specific periodization as is common, for example, in athletes [88], where dividing training into consecutive phases with specific objectives based on training period/season is common [70]. While the studied combinations of strength and endurance training appeared to consistently result in improvements in maximal strength and other characteristics that contribute/or may contribute to endurance capacity/performance, further combinations and approaches to programming may need to be explored. Indeed, the combinations of strength and endurance training used in included studies may not have employed a training load or frequency high enough and/or training interventions that were long enough to induce significant changes in maximal strength, fast-force production, endurance performance/capacity, or an “interference effect.”

The endurance training modes utilized in the included studies were overwhelmingly running or cycling, although several other endurance training modes may be used by physically active and athletic females. Endurance training modes such as running and cycling employ different force-production strategies [89–91] that should be taken into consideration when evaluating training adaptations. Running requires repetitive and relatively fast force production using the stretch–shortening cycle [92], where even maximal uphill running does not elicit maximal muscle activation of the lower extremities [93]. Cycling requires prolonged repeated force production [91]. In addition, the quadriceps femoris and gluteal muscles play a more significant role in cycling than in running, where the biceps femoris is more involved [94]. Rowing and cross-country skiing are important endurance training modes that employ the upper body, which is often overlooked in research on concurrent strength and endurance training and females, in general. Included studies primarily focused on training the lower extremities, where only three studies included upper body strength training and testing (as a part of whole body training or upper body training alone (Hoff et al. [85] in cross-country skiers as well as Haykowsky et al. [84] and Bell et al. [39] in rowers). Females generally have lower upper body strength than males [95, 96], and more research regarding concurrent strength and endurance training in the upper body is warranted in all populations that require efficient use of the upper body for performance. Endurance performance was primarily assessed using sport-specific *V*O_2max_ tests, although field tests to assess endurance performance were also utilized. It is important to remember that laboratory tests may not be fully representative of sport-specific or functional performance requirements; thus, caution should be used when interpreting results.

The studies in this review included “hypertrophic” or maximal and/or explosive/plyometric training (or a progression from “hypertrophic” towards maximum and explosive/plyometric training) ~ 2–3 times per week combined with running, cycling, rowing, or cross-country skiing up to 4 times per week. The strength training loads employed varied considerably, ranging from 2 to > 10 repetitions per 2–6 sets. Improvements in maximal strength were assessed primarily using bilateral 1-RM leg press or squat. Again, these types of laboratory tests for maximal strength may not be fully representative of sport-specific or functional performance requirements; thus, caution should be used when interpreting results.

Fast-force production, also known as the rate of force development or the ability of the neuromuscular system to generate force rapidly particularly early in rapid contractions, is essential for performance in fundamental movements needed for sports performance such as jumping, throwing, and sprinting [9]. Indeed, the fast-force production appears to be linked to sport-specific and functional daily tasks and is more sensitive for detecting changes in neuromuscular function than maximal strength testing [97], although fast-force production in females is an understudied topic [98]. We might expect that concurrent strength and endurance training increases fast-force production in females, because countermovement jump (height, peak power, and peak velocity) correlates well with measures of maximal strength like 1-RM squat and power clean [99], which generally appear to improve as a result of concurrent strength and endurance training. This is supported by Hendrickson et al. [74], who reported similar increases in squat jump peak power (watts) between females performing strength training and females performing concurrent strength and endurance training, and Barnes et al. [79], who reported that peak force from a straight-leg 5-jump plyometric jump test (Nkg^−1^) was larger in females combining running with heavy resistance training than in females performing volume-matched plyometric and heavy resistance training. Regrettably, further analysis of the influence of concurrent strength and endurance training on fast-force production in females is limited by a lack of data.

### The “Interference Effect”

Athletes/exercisers and coaches/personal trainers may worry about endurance training interfering with strength development and fast-force production [7, 8] and/or muscle hypertrophy [10] or muscle hypertrophy “interfering” with endurance performance; however, the present literature does not appear to substantiate these worries in females. Regrettably, the presence of an “interference effect” and/or potential mechanism behind this “interference effect” are difficult to evaluate in female participants as only six of the included studies employed study designs that might be used to assess “chronic interference” [39, 74–77, 85]. Two studies can be used to examine the influence of concurrent strength and endurance training in comparison to strength and endurance training alone [74, 75], three studies can assess the differences or the possibility of “interference” of concurrent training on endurance performance [76, 77, 85], and only one study can assess the possibility of “interference” of concurrent training on strength development [39]. Importantly, the primary aim of these six studies was not to explore “interference,” and none of the included studies demonstrated clear disadvantages for strength or endurance adaptations when strength and endurance training were performed concurrently. Instead, strength training added to endurance training improved strength and running economy [76], although observed improvements in strength were not reflected in increased endurance performance more than endurance training alone [77]. In the context of cross-country skiing and rowing, upper body endurance training combined with maximal whole body strength training improved work economy/endurance performance more effectively than endurance or strength training alone [39, 85]. The only studies that compared strength only, endurance only, concurrent strength and endurance, and control groups were Nindl et al. [75] and Hendrickson et al. [74]. These studies demonstrated “specificity of training,” where strength training significantly improved maximal strength and endurance training significantly improved endurance capacity. More specifically, Hendrickson et al. [74] demonstrated that concurrent strength and endurance training improved strength performance at comparable levels to strength training alone, while also demonstrating that concurrent strength and endurance training improved endurance capacity at comparable levels to endurance training alone. Collectively, these results suggest an absence of an “interference” effect, as performing strength and endurance training concurrently did not appear to be less advantageous than strength or endurance training alone. Due to the limited data available, sweeping conclusions regarding the presence or absence of “interference” in apparently healthy female populations cannot be made. Moreover, the heterogeneity in training background and training combinations in addition to the relatively lower volume of overall training make it impossible to draw any conclusions about “interference” in apparently healthy females.

A unique approach to concurrent strength and endurance research was used by Eklund et al. [82, 83] and Schumann et al. [81], who examined whether or not acute “interference” due to the order of exercise, i.e., endurance before strength or strength before endurance, on the same day and in the same session versus performing strength and endurance on separate days might result in chronic “interference.” Eklund et al. [82] reported that endurance before strength, strength before endurance, and different-day concurrent training were all effective in improving measures of maximal strength and endurance capacity, but that different-day concurrent training induced a greater magnitude of improvement in endurance performance than same-day endurance followed by strength or strength followed by endurance. Likewise, training endurance before strength or strength before endurance on the same day yielded similar increases in muscle cross-sectional area [83]. Additionally, Schumann et al. [81] reported that endurance before strength, strength before endurance, and different-day strength and endurance training improves endurance capacity in both females and males, while females appear to have additional improvements in submaximal endurance capacity when endurance training is performed before strength training on the same day.

Total training volume and the ratio of strength training to endurance training influences training adaptations. In general, a higher volume of endurance training in combination with strength training is associated with “interference” in males [100]. The studies included in this review employed a relatively low volume and frequency of concurrent strength and endurance training. Likewise, the duration of training interventions may influence “interference” or a lack thereof. Shorter interventions, such as those utilized in the included studies, may not reveal “interference,” particularly in untrained populations [101] and especially if training frequency is low [102, 103]. Medium-length or so-called prolonged interventions (lasting, e.g., 9–12 or 13–24 weeks, respectively) are more likely to reveal “interference,” especially if training frequency/volume is high [8, 104]. While the included studies ranged in duration from 7 to 24 weeks and included 1–3 strength training sessions per week, most of the studies lasted between 9 and 16 weeks and all of the 24-week studies were from the same laboratory. Furthermore, as previously mentioned, the frequency and volume of strength and endurance training remained low to moderate in all of the included studies, thus limiting our ability to evaluate the potential for “interference” in situations where a higher volume and intensity of training are used.

Finally, training status also influences susceptibility to “interference.” Studies included in this review investigate concurrent strength and endurance training in a relatively heterogeneous population including participants that were untrained, recreationally trained, and trained [73]. When endurance training is added to strength training, it may lead to positive adaptations in strength in moderately trained and untrained individuals, but in trained individuals, the influence may even be negative [18]. On the other hand, untrained individuals may be more sensitive to physiological stress than trained individuals [8], where starting with low to moderate training frequency and volume would be recommended.

### Quality of Included Studies and Limitations

The current review identified only a limited number of studies investigating concurrent strength and endurance training studies in females. It is possible that some studies were overlooked during the review process due to searching only two databases. Training studies are understandably a challenging undertaking; many laboratories do not have adequate resources to complete long-term and/or well-controlled training studies. The quality of the included studies ranged from low to moderate, with the majority receiving a score indicating “moderate” quality. Several studies lacked power calculations and did not report compliance with the intervention or whether participants were randomized into intervention groups. The group size for the included studies was consistently < 20, which may have influenced the statistical power as well as the generalizability of results. Importantly, the a priori inclusion criteria employed in the present review excluded several studies (see Fig. 1). The lack of consideration and/or reporting of menstrual status and hormonal contraceptive use should be considered in future research.

### Future Directions and Female-Specific Considerations in Concurrent Strength and Endurance Training

It is worth noting that most of the included studies did not take into consideration or report menstrual status or hormonal contraceptive use, which may be among the limiting factors in understanding adaptations to concurrent strength and endurance training (as well as adaptations to training in general) in females. Indeed, exercise testing and research has often been completed with little or no consideration of hormonal profiles, while some research has, undoubtedly, been unfulfilled due to the potential “confounding factors” incurred by the hormonal fluctuations or use of exogenous hormones that are a significant part of the lives of most females of “reproductive age.” The most recent meta-analyses indicate that the effect of the menstrual cycle on performance is only trivial [105], while the influence of hormonal contraceptive use on training adaptations is relatively small [72, 106], Overall, however, studies examining the influence of the menstrual cycle on performance or influence of hormonal contraceptives on training adaptations are of low quality. Thus, unequivocal conclusions regarding the influence of hormonal profile on performance cannot be made. Importantly, the individual effects of hormonal profiles on training, while not necessarily statistically significant, may be meaningful for individual athletes [107].

A growing body of research, which has been primarily performed in females, indicates that low energy availability could be a factor in the observed plateaus and/or decreases in performance and health (including bone density) [108, 109]. We hypothesize that observed plateaus and decreases in performance that have been described as or identified as “interference” might be explained, in part, by hormonal dysfunction related to inadequate energy availability. Regrettably, the current body of concurrent strength and endurance training research does not control for, or monitor, e.g., menstrual status or energy availability during training interventions. Energy availability is considered a prerequisite for both high-quality training sessions and recovery [110–112], where even short-term deficits in energy availability can result in decreased muscle protein synthesis [113, 114] and short- to medium-term deficits in energy availability can blunt training response and/or impair recovery, thus predisposing athletes to undesired overreaching or overtraining [67, 115–117]. To advance female exercise physiology and sport science research, methodology including participant selection, experimental design, and exploration of hormonal profiles, and, for example, confirmation of hormonal status (menstrual status and hormonal contraceptive use) should be considered to further elucidate the diversity and complexities associated with female physiology [118, 119]. In practice, simply taking into consideration and recording of the menstrual cycle status/phase/hormonal contraceptive use may help to explain more about increases, decreases, and plateaus in training adaptations that could be related to hormonal status.

Finally, while not a focus of this paper, programming concurrent strength and endurance according to the phases of the menstrual cycle has been promoted in popular and social media, and it is worth mentioning that the present review found no evidence to support this approach. While some research has indicated that periodizing strength training according to menstrual cycle phase (e.g., higher volume training during the follicular phase than in the luteal phase) might be beneficial for increasing strength and muscle mass in the lower extremities [120–123], these benefits are not reported in the upper extremities [124]. Furthermore, no training studies exist to date that demonstrate benefits from periodizing endurance training according to menstrual cycle phase, although cross-sectional studies indicate an increased dependency on fat oxidation during the luteal phase [125, 126]. Lastly, there are no training studies published to date that would indicate any physiological advantages from programming or planning concurrent strength and endurance training by menstrual cycle phase.

## Conclusions

Concurrent strength and endurance training appears to improve strength and endurance capacity in female populations. However, there are several research paradigms that still need to be explored, such as the “interference” effect in athletic female populations, the effects of concurrent strength and endurance training on fast-force production in females, and the effect of menstrual status and hormonal contraceptive use (hormone profiles) on concurrent strength and endurance training adaptations. Additionally, the influence of time of day (chronobiology) on concurrent strength and endurance training outcomes (e.g., [127]) is yet to be investigated in females.

A meta-analysis of concurrent strength and endurance training in females is premature due to the limited volume of concurrent strength and endurance training research in females. Furthermore, a meta-analysis of such a heterogeneous group of studies may not accurately reflect the efficacy of concurrent strength and endurance training [128]. Likewise, it is difficult to draw strong conclusions or generalizations regarding specific combinations of concurrent strength and endurance training in females, due to the heterogeneous fitness levels, training plans, and training durations presented in the included literature. Indeed, several challenges exist in the area of concurrent strength and endurance training research, as a plethora of training combinations exist (and are constantly added). Different approaches to training may reveal subtle, but meaningful, differences in training responses and adaptations. As such, and based on the included literature, evidence-based modifications to specific exercise prescription for females cannot be made, although it may be suggested the “more important” training mode be completed first when combining strength and endurance into the same session [81] and that concurrent strength and endurance training may be most effective (in recreationally active populations) when performed on separate days [18, 81, 82]*.* Additional exploration of sport-specific concurrent strength and endurance training, including the upper body, would be useful for practitioners making evidence-based decisions regarding testing and training for some athlete populations. Furthermore, future research about concurrent strength and endurance training in females should consider hormone profiles including menstrual status (energy availability [67]) and hormonal contraceptive use [62–64] and potentially also the reason for hormonal contraceptive use.

### Supplementary Information

Below is the link to the electronic supplementary material.

Supplementary file1 (PDF 114 kb)

## Declarations

### Availability of data and material

The datasets used and/or analyzed during the current study are available from the corresponding author on reasonable request.
