# Supplementary material for: Perspectives on Concurrent Strength and Endurance Training in Healthy Adult Females: A Systematic Review
Source: Sports Med. 2023 Nov 10;54(3):673–96. doi: 10.1007/s40279-023-01955-5 (PMC10978686; doi:10.1007/s40279-023-01955-5)
Supplement: Supplementary file 1 — Supplementary file1 (PDF 114 kb) [file 40279_2023_1955_MOESM1_ESM.pdf]

APPENDIX 1

Concurrent strength and endurance training in women of reproductive age: A Systematic Narrative Review. Corresponding author: Dr Ritva S. Mikkonen, Sports Technology Unit, Faculty of Sport and Health Sciences, University of Jyväskylä, Vuokatti, Finland. E-mail: ritva.s.taipale@jyu.fi

Electronic Supplementary Material: Example of Search Strategy Conducted in PubMed July 6, 2021.

**LIMITS APPLIED**	
Human<br>Publications in English<br>Full text	
**SEARCH TERMS**	
Combined strength and endurance training AND women	136
Concurrent strength and endurance training AND women	34
Combined strength and endurance training AND female*	412
Concurrent strength and endurance training AND female*	90
Combined resistance and endurance training AND women	112
Concurrent resistance and endurance training AND women	27
Combined resistance and endurance training AND female*	298
Concurrent resistance and endurance training AND female*	73
Combined resistance and aerobic training AND women	609
Concurrent resistance and aerobic training AND women	88
Combined resistance and aerobic training AND female*	1,622
Concurrent resistance and aerobic training AND female*	219
Combined strength and aerobic training AND women	585
Concurrent strength and aerobic training AND women	73
Combined strength and aerobic training AND female*	1,699
Concurrent strength and aerobic training AND female*	230
**Total: 6307 (including duplicates)**	

Electronic Supplementary Material: Example of Search Strategy Conducted in Ovid MEDLINE July 6, 2021.

LIMITS APPLIED	
Human<br>Publications in English<br>Full text	
**SEARCH TERMS**	
Combined strength and endurance training AND women	8
Concurrent strength and endurance training AND women	7
Combined strength and endurance training AND female*	12
Concurrent strength and endurance training AND female*	9
Combined resistance and endurance training AND women	1
Concurrent resistance and endurance training AND women	2
Combined resistance and endurance training AND female*	2
Concurrent resistance and endurance training AND female*	2
Combined resistance and aerobic training AND women	3
Concurrent resistance and aerobic training AND women	0
Combined resistance and aerobic training AND female*	6
Concurrent resistance and aerobic training AND female*	0
Combined strength and aerobic training AND women	1
Concurrent strength and aerobic training AND women	0
Combined strength and aerobic training AND female*	2
Concurrent strength and aerobic training AND female*	1
**Total: 56 (including duplicates)**	

Electronic Supplementary Material: Example of Search Strategy Conducted in PubMed December 16, 2022.

LIMITS APPLIED	
Human<br>Publications in English<br>Full text<br>Published between January 2021 December 2022	
**SEARCH TERMS**	
Combined strength and endurance training AND women	16
Concurrent strength and endurance training AND women	3
Combined strength and endurance training AND female*	35
Concurrent strength and endurance training AND female*	12
Combined resistance and endurance training AND women	13

Concurrent resistance and endurance training AND women	2
Combined resistance and endurance training AND female*	28
Concurrent resistance and endurance training AND female*	6
Combined resistance and aerobic training AND women	130
Concurrent resistance and aerobic training AND women	20
Combined resistance and aerobic training AND female*	231
Concurrent resistance and aerobic training AND female*	35
Combined strength and aerobic training AND women	119
Concurrent strength and aerobic training AND women	13
Combined strength and aerobic training AND female*	0
Concurrent strength and aerobic training AND female*	0
**Total: 663 (including duplicates)**	

APPENDIX 2

Concurrent strength and endurance training in women of reproductive age: A Systematic Narrative Review. Corresponding author: Dr Ritva S. Mikkonen, Sports Technology Unit, Faculty of Sport and Health Sciences, University of Jyväskylä, Vuokatti, Finland. E-mail: ritva.s.taipale@jyu.fi

Electronic Supplementary Material: Study Quality Assessment

**Downs and Black checklist (maximum attainable score = 16)**<br><br>**Study quality was classified as follows:**<br>***"High" = 14 – 16***<br>***"Moderate" = 10 – 13***<br>***"Low" = 6 – 9***<br>***"Very low" = 0 – 5***	
**REPORTING**	
Q1	**Is the hypothesis/aim/objective of the study clearly described?**<br>Yes = 1<br>No = 0
Q2	**Are the main outcomes to be measured clearly described in the Introduction or Methods section?** *If the main outcomes are first mentioned in the Results section, the question should be answered no.*<br>Yes = 1<br>No = 0
Q3	**Are the characteristics of the patients included in the study clearly described?** *In cohort studies and trials, inclusion and/or exclusion criteria should be given. In case-control studies, a case-definition and the source for controls should be given.*<br>Yes = 1<br>No = 0
Q4	**Are the combined strength and endurance training interventions clearly described?** *Training interventions and control group activity (where relevant) should be clearly described.*<br>Yes = 1<br>No = 0
Q5	**Are the main findings of the study clearly described?** *Simple outcome data should be reported for all major findings so that the reader can check the major analyses and conclusions. (This question does not cover statistical tests which are considered below).*<br>Yes = 1<br>No = 0
Q6	**Does the study provide estimates of the random variability in the data for the main outcomes?** *In non-normally distributed data the inter-quartile range of results should be reported. In normally distributed data the standard error, standard deviation or confidence intervals should be reported. If the distribution of the data is not described, it must be assumed that the estimates used were appropriate and the question should be answered yes.*<br>Yes = 1<br>No = 0
**EXTERNAL VALLIDITY**	
Q7	**Were the participants' hormonal status (menstrual status or hormonal contraceptive use) assessed and/or confirmed as part of the study design?**<br>Yes = 1<br>No = 0<br>Unable to determine = 0

INTERNAL VALIDITY - BIAS	
Q8	**Was at least one familiarization trial conducted prior to exercise testing?**<br>Yes = 1<br>No = 0<br>Unable to determine = 0
Q9	**Were the exercise test conditions adequately standardised (taking into consideration factors including time of day, prior nutritional intake [including caffeine] and prior exercise)?**<br>Yes (all relevant factors standardised) = 2<br>Yes (some relevant factors standardised) = 1<br>Exercise testing unstandardized = 0<br>Unable to determine = 0
Q10	**If any of the results of the study were based on "data dredging", was this made clear?** *Any analyses that had not been planned at the outset of the study should be clearly indicated. If no retrospective unplanned subgroup analyses were reported, then answer yes.*<br>Yes = 1<br>No = 0<br>Unable to determine = 0
Q11	**Were the statistical tests used to assess the main outcomes appropriate?** *The statistical techniques used must be appropriate to the data. For example non-parametric methods should be used for small sample sizes. Where little statistical analysis has been undertaken but where there is no evidence of bias, the question should be answered yes. If the distribution of the data (normal or not) is not described, it must be assumed that the estimates used were appropriate and the question should be answered yes.*<br>Yes = 1<br>No = 0<br>Unable to determine = 0
Q12	**Were the main outcome measures used accurate (valid and reliable)?** *For studies where the outcome measures are clearly described, the question should be answered yes. For studies which refer to other work or that demonstrates the outcome measures are accurate, the question should be answered as yes.*<br>Yes = 1<br>No = 0<br>Unable to determine = 0
**INTERNAL VALIDITY – CONFOUNDING (SELECTION BIAS)**	
Q13	**Was assignment to training group randomized?**<br>Yes = 1<br>No = 0<br>Unable to determine = 0
**POWER**	
Q14	**Did the study have sufficient power to detect an *a priori* specified scientifically important effect at a pre-determined probability threshold?** Answer yes if they included a power calculation, and no if not.<br>Yes = 1<br>No = 0
Q15	**Was study retention > 85%?**<br>Yes = 1<br>No = 0<br>Unable to determine = 0
